# Measurement of cations, anions, and acetate in serum, urine, cerebrospinal fluid, and tissue by ion chromatography

**DOI:** 10.14814/phy2.13666

**Published:** 2018-04-13

**Authors:** Andrew D. Chapp, Simeon Schum, Jessica E. Behnke, Taija Hahka, Michael J. Huber, Enshe Jiang, Robert A. Larson, Zhiying Shan, Qing‐Hui Chen

**Affiliations:** ^1^ Department of Kinesiology and Integrative Physiology Michigan Technological University Houghton Michigan; ^2^ Department of Biological Sciences Michigan Technological University Houghton Michigan; ^3^ Department of Chemistry Michigan Technological University Houghton Michigan; ^4^ Department of Internal Medicine Carver College of Medicine University of Iowa Iowa City Iowa

**Keywords:** Anion, cations, electrolyte, ion chromatography

## Abstract

Accurate quantification of cations and anions remains a major diagnostic tool in understanding diseased states. The current technologies used for these analyses are either unable to quantify all ions due to sample size/volume, instrument setup/method, or are only able to measure ion concentrations from one physiological sample (liquid or solid). Herein, we adapted a common analytical chemistry technique, ion chromatography and applied it to measure the concentration of cations; sodium, potassium, calcium, and magnesium (Na^+^, K^+^, Ca^2+^, and Mg^2+^) and anions; chloride, and acetate (Cl^−^, ^−^ OAc) from physiological samples. Specifically, cations and anions were measured in liquid samples: serum, urine, and cerebrospinal fluid, as well as tissue samples: liver, cortex, hypothalamus, and amygdala. Serum concentrations of Na^+^, K^+^, Ca^2+^, Mg^2+^, Cl^−^, and ^−^ OAc (mmol/L): 138.8 ± 4.56, 4.05 ± 0.21, 4.07 ± 0.26, 0.98 ± 0.05, 97.7 ± 3.42, and 0.23 ± 0.04, respectively. Cerebrospinal fluid concentrations of Na^+^, K^+^, Ca^2+^, Mg^2+^, Cl^−^, and ^−^ OAc (mmol/L): 145.1 ± 2.81, 2.41 ± 0.26, 2.18 ± 0.38, 1.04 ± 0.11, 120.2 ± 3.75, 0.21 ± 0.05, respectively. Tissue Na^+^, K^+^, Ca^2+^, Mg^2+^, Cl^−^, and ^−^ OAc were also measured. Validation of the ion chromatography method was established by comparing chloride concentration between ion chromatography with a known method using an ion selective chloride electrode. These results indicate that ion chromatography is a suitable method for the measurement of cations and anions, including acetate from various physiological samples.

## Introduction

The measurement of electrolyte concentrations in physiological samples remains an important diagnostic tool to many disease states. Common cations and anions measured include: sodium, potassium, magnesium, calcium, and chloride. Typically, the choice measurement methods are, flame photometry (Hald 1947) or ion selective electrodes (Fogh‐Andersen et al. 1984; Levy 1981). While both of these methods give consistent and reproducible results, they are limited in that they are usually exclusive for single ions per sample and require larger sample volumes. Furthermore, these measurements are typically confined to liquid samples, that is, cerebrospinal fluid and/or serum/plasma. Tissue measurements usually involve radiolabeled nuclei (Christensen et al. 1996; Green et al. 1955) or more recently, magnetic resonance spectroscopy (Christensen et al. 1996; Kopp et al. 2012; Reetz et al. 2012; Wang et al. 2013; Yushmanov et al. 2007), although other techniques such as ashing followed by flame photometry have been implemented (Titze et al. 2005, 2002).

The necessity in measurement of cations and anions spans a vast array of physiological conditions, ranging from: hypertension (Foss et al. 2013; Gomes et al. 2017; Kopp et al. 2012; Lohmeier et al. 2005; Osborn et al. 2012; Stocker et al. 2015), water and electrolyte homeostasis (Mack et al. 1994; Nose et al. 1988; Schreihofer et al. 1999), stroke (Chung et al. 2015; Rappaport et al. 1987), and alcohol intoxication (Avery et al. 1983; Liamis et al. 2000; Tonisson et al. 2010) to name a few. Additionally, these traditional techniques exclude small organic ions which can aid in diagnosis and understanding disease progression such as in cancer (Hirschhaeuser et al. 2011; Kamphorst et al. 2014; Semenza 2008).

For small organic ions such as acetate, the typical method is that of an ester derivatization(Perry et al. 2016; Richards et al. 1975; Tumanov et al. 2016; Turnbaugh et al. 2006). This method requires an ester derivatizing agent, strong acids/bases and organic extractions followed by analysis on gas chromatography. The drawback to this technique is, (1) incomplete chemical conversion to the ester, (2) competing reactions of other carboxylic acid functional groups (lactate/lactic acid, carboxyl functional groups on free amino acids, etc.) with the ester converting reagent (Fig. 9) and (3) incomplete extraction of the ester into the organic phase for GC analysis. A previous ester derivatization technique from Richards and colleagues reported a 15% extraction efficiency using liver tissue and serum (Richards et al. 1975). The use of earlier IC systems explored biological samples (Rich et al. 1980), however, these methods were more tedious and were often utilized without ion suppression, which is now common on newer IC systems. This method demonstrates the use of ion chromatography for the accurate measurement of ions from physiological samples, including both liquid and tissue samples obtained from Sprague‐Dawley rats. Preparation of solid samples through sonication and aqueous extraction reduce the need for derivatizing agents, strong acids/bases and organic extractions for organic anions such as acetate, and are also capable of extracting other ions as well. Minimal sample volume 10 *μ*L, diluted 1000 fold, make this an ideal choice for multi‐ion analysis from physiological samples. If equipped with a splitter, cations, anions, and small organic ions can all be measured simultaneously from one sample.

## Materials and Methods

### Reagents

Glacial acetic acid was obtained from Sigma‐Aldrich (St. Louis, MO) and ultra‐pure water (>18 MΩ) obtained in‐house by filtration and purification of house distilled water from an EasyPure II water filtration system (Barnstead). Methanesulfonic acid (Alfa Aesar) anion and cation standards were obtained from ThermoScientific.

### Animals

Male Sprague‐Dawley (SD) rats purchased from Charles River Labs (Wilmington, MA) were individually housed in a temperature controlled room (22–23°C) with a 14:10 h. light:dark cycle. Chow and tap water were available ad libitum unless otherwise noted. All experimental and surgical procedures were carried out under the guidelines of the National Institutes of Health Guide for the Care and Use of Laboratory Animals with the approval of the Institutional Animal Care and Use Committee at Michigan Technological University.

### Normal salt urine collection

Normal salt (NS) (0.4%) feed was purchased from Harlan Labs, (Harlan, IN). SD rats were given a NS diet for 21 days with normal drinking water. After 21 days they were transferred to metabolic cages and housed individually. The rats were provided with the same consistent diet while in the metabolic cages and were given 24 h to acclimate to their new environment. Following 24 h acclimation, any urine collected was discarded prior to metabolic measurements. Metabolic measurements and urine collection were performed on the second, third, and fourth days consecutively. Urine samples were centrifuged for 10 min and 1 mL of the supernatant was collected and stored at −80°C until analysis.

### Tissue and liquid sample collection

Male Sprague‐Dawley rats 300–550 g were given an intraperitoneal (I.P.) injection of sodium pentobarbital (50 mg/kg body weight) and cerebral spinal fluid was collected through a needle puncture in the cisterna magna as previously described (Nirogi et al. 2009). Then, venous blood was taken through a right ventricle draw. The brain was rapidly removed and a sample of tissue from each brain region; cortex, hypothalamus, and amygdala were placed into separate 1.5 mL centrifuge tubes and flash frozen in liquid nitrogen. Liver tissue was harvested similarly to brain tissue. Blood samples were centrifuged at 13,000 rcf for 10 min and the serum was removed to a fresh 1.5 mL centrifuge tube. All samples were then stored in a −80°C freezer until analysis by ion chromatography.

### Sample preparation

**Figure 1:**
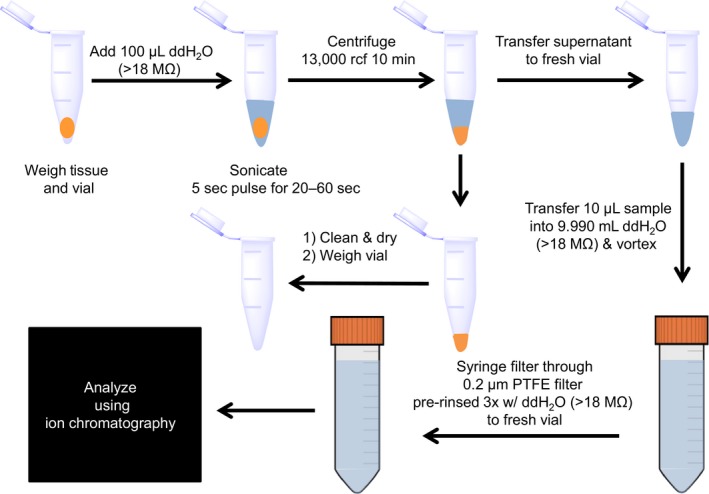
Flow diagram for sample preparation. Tissue samples are weighed in their vials, and 100 *μ*L ddH_2_O (>18 MΩ) added. Samples are sonicated for 20–60 sec in 5 sec pulses at 20 kHz or until well homogenized. The samples are centrifuged at 13,000 rcf for 10 min and the supernatant removed and transferred to a fresh 1.5 mL centrifuge tube. Empty tissue vials are washed, dried, and weighed to determine the mass of tissue. Liquid samples are prepared by adding 10 *μ*L sample with 9.990 mL ddH_2_O (>18 MΩ), vortexed and syringe filtered into fresh vials. Samples are then run on ion chromatography to determine cation and anion concentrations.

For tissue samples (Fig. 1); samples were thawed to room temperature and the centrifuge tube containing the tissue was weighed on an analytical balance before and after extraction. Tubes after extraction were washed with water, followed by ethanol (70%) and allowed to completely dry before weighing. For the extraction, 100 *μ*L of ddH_2_O (>18 MΩ) was added to the tube and the tissue was sonicated in pulses of 5 sec at 20 kHz for a total of 20–60 sec or until the tissue was well homogenized. Tissue samples were centrifuged at 13,000 rcf for 10 min and the supernatant was removed to a fresh 1.5 mL centrifuge tube. The liquid sample extracts (Fig. 1) were then diluted 1000 fold by adding 10 *μ*L liquid sample to 9.990 mL of ddH_2_O (> 18 MΩ) in sterile 15 mL centrifuge tubes. Liquid samples of CSF and serum were diluted 1000 fold for ion chromatography by adding 10 *μ*L liquid sample to 9.990 mL of ddH_2_O (>18 MΩ) in sterile 15 mL centrifuge tubes. All diluted samples were filtered through preflushed (3X, ddH_2_O, >18 MΩ) sterile PTFE syringe filters (0.2 *μ*m) and sterile 3 mL disposable syringes (BD) into new, sterile 15 mL centrifuge tubes. The samples were transferred to ion chromatography vials (5 mL, Thermofisher) and analyzed via ion chromatography (Dionex ICS‐2100).

### Measurement and dual detection of cations and anions, IC

Diluted and filtered samples were added to individual sample poly vials (5 mL, Thermofisher) and loaded into a Dionex AS‐DV autosampler (Thermofisher) connected to a splitter valve. From the splitter valve, identical length high pressure tubing was connected to a Dionex ICS‐2100 (anions) and Dionex ICS‐1100 (cations) ion chromatography systems (Thermofisher). 1 mL of sample was injected from the poly vial and split to 0.5 mL which was passed to each IC system.

### Sodium, potassium, calcium, and magnesium measurements, IC

For cations, the Dionex 1100 was equipped with a CS12A 4 mm analytical and a Dionex IonPac CG12A 4 mm guard column set. 20 mmol/L MSA was the eluent and was sonicated for 20 min followed by degassing with nitrogen for an additional 10 min prior to IC. The sample was eluted for 15.5 min with isocratic 20 mmol/L methanesulfonic acid. Cation concentrations were determined from standard curves for each cation of interest. Unknown sample cation concentrations were determined based off the linear fit and then back calculated based on the 1000 fold dilution. For tissue samples, the concentration obtained from the linear fit was back calculated for the 1000 fold dilution, converted from concentration to moles and then divided by the tissue sample weight in volume to give the final tissue concentration.

### Chloride and acetate measurements, IC

For anions, the Dionex 2100 was equipped with a Dionex EGC III KOH RFIC, potassium hydroxide (KOH) eluent generator cartridge (Thermofisher) and an AS17‐C 4 mm analytical and guard column set. Water (>18 MΩ) used for generating the eluent was sonicated for 20 min followed by degassing with nitrogen for an additional 10 min prior to IC. The sample was eluted with KOH using the following method.

1. 5–0 min: Equilibration at 1 mmol/L KOH.
2. 0–5 min: Isocratic at 1 mmol/L KOH.
3. 5–15 min: Ramp, 1–30 mmol/L KOH.
4. 15–20 min: Ramp, 30–40 mmol/L KOH

**Figure 2:**
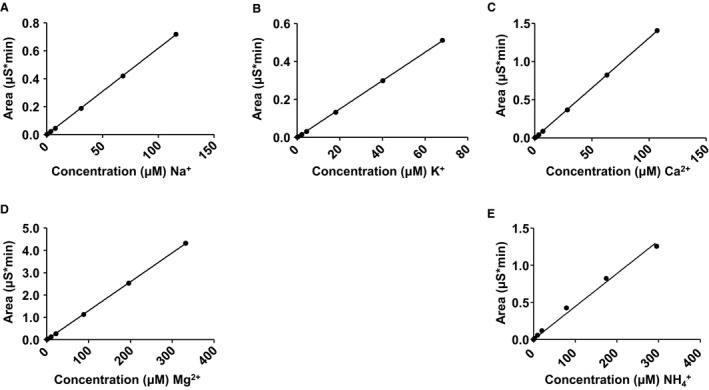
Cation standard curves. (A) Sodium (Na^+^) standard curve. (B) Potassium (K^+^) standard curve. (C) Calcium (Ca^2+^) standard curve. (D) Magnesium (Mg^2+^) standard curve. (E) Ammonium (NH_4_^+^) standard curve. A linear regression line was fitted to each standard curve and unknown cation concentrations determined from the slope of the line.

To determine the acetate concentration from the samples, a standard curve was constructed for known concentrations of acetate and a linear regression line was used from the areas under the curve for the known concentrations of acetate (Fig. 2A). Unknown sample acetate concentrations were determined based off the linear fit and then back calculated based on the 1000 fold dilution. For tissue samples, the concentration obtained from the linear fit was back calculated for the 1000 fold dilution, converted from concentration to moles and then divided by the tissue sample weight in volume to give the final tissue concentration.

### Chloride measurements, ion selective electrode (ISE)

As a proof of concept, chloride concentration measurements from CSF in SD rats were also analyzed using a commercially available, ion selective electrode device for medical, diagnostic use (Ortho Vitros 5600). Samples were prepared and run according to the manufacturer's instructions on a chloride selective slide (Vitros). Briefly, prior to sample analysis, a four point calibration curve was created (Vitros Calibrator Kit 2), followed by two control chloride samples (Vitros). 10 *μ*L CSF and 10 *μ*L reference (Vitros Reference Fluid) were placed on separate halves of the slide resulting in a migration of both fluids toward the center of the slide where a paper bridge is located. A stable liquid junction potential was obtained which connects the sample electrode to the reference electrode. The potential difference between each electrode is equivalent to the chloride concentration in the sample, in this case the CSF.

### Statistical analysis

Data values were reported as mean ± SEM. Depending on the experiments, group means were compared using either unpaired Student's *t*‐test, or a one‐way ANOVA. Differences between means were considered significant at *P *<* *0.05. Where differences were found, Bonferroni post hoc tests were used for multiple pair‐wise comparisons. All statistical analyses were performed with a commercially available statistical package (GraphPad Prism, version 5.0).

## Results

The aim of this study was to employ a method for the concentration measurement of ions obtained from physiological samples, both liquid and solid, for the application in diagnostics in diseased states. Since many samples obtained in research settings from animals and cell culture are small in either volume or size, having an accurate and reliable method for quantifying ion concentrations, including acetate is of utmost importance. Thus, we adapted a known analytical chemistry instrument, ion chromatography and developed a method for extracting and measuring cations, anions, and acetate.

### Importance of clean tubes and fresh ultra‐pure water for acetate measurements

What we found, consistent with other groups (Richards et al. 1975; Tumanov et al. 2016), is that there is heavy acetate contamination in water supplies, and even ultra‐pure water (>18 MΩ), overtime begins drawing in organics that contaminate the water used for sample preparation. We therefore obtained fresh ultra‐pure water (>18 MΩ) prior to sample extractions or sample preparation. Furthermore, sterile, cell culture grade centrifuge tubes, syringes, and filters, also contained heavy acetate contamination. Centrifuge tubes were sonicated for 1 h in ultra‐pure water (>18 MΩ) prior to sample preparation and thoroughly dried. Syringes and filters were flushed at least 3× with ultra‐pure water prior to sample filtration. This diligence reduced acetate contamination in blank samples (negative control) to undetectable levels, which have not been previously reported in other methodologies for acetate measurements (Richards et al. 1975; Tumanov et al. 2016).

### Cation standard curve and representative IC chromatograms

**Figure 3:**
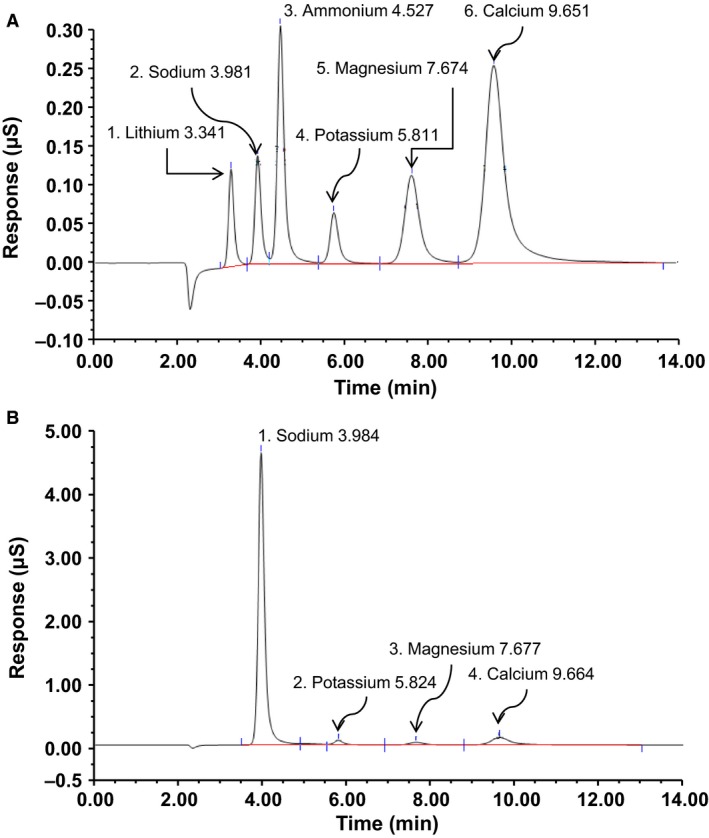
Representative cation chromatograms from IC. (A) Representative cation standards chromatogram containing lithium (Li^+^), sodium (Na^+^), ammonium (NH_4_^+^), potassium (K^+^), magnesium (Mg^2+^), and calcium (Ca^2+^). (B) Representative cation chromatogram from a cerebrospinal fluid (CSF) sample obtained from a Sprague‐Dawley rat.

We first prepared standard curves for all cations of interest, Na^+^, K^+^, Ca^2+^, Mg^2+^, and NH_4_^+^_._ Various concentrations of the cations were prepared and linear regression lines for each cation of interest constructed (Fig. 2). Regression lines were well fit and utilized to determine unknown cation concentrations from liquid and tissue samples. Chromatograms from each injection were analyzed for their area under the curve either for the standard curves or from liquid or tissue samples. Figure 3A is a representative chromatogram for a cation standard which includes all cations of interest. Elution and detection for the following cations (Li^+^, Na^+^, NH_4_^+^, K^+^, Mg^2+^, and Ca^2+^) were well resolved and occurred at the following times, post injection (min): 3.341, 3.981, 4.527, 5.811, 7.674, and 9.651, respectively. Unknown samples had similar elution and detection times if the cation was present. Figure 3B is a representative sample trace from a CSF sample containing the following cations (Na^+^, K^+^, Mg^2+^, and Ca^2+^) with elution and detection times, post injection (min): 3.984, 5.824, 7.677, and 9.664, respectively.

### Cation concentrations in liquid and tissue samples

**Figure 4:**
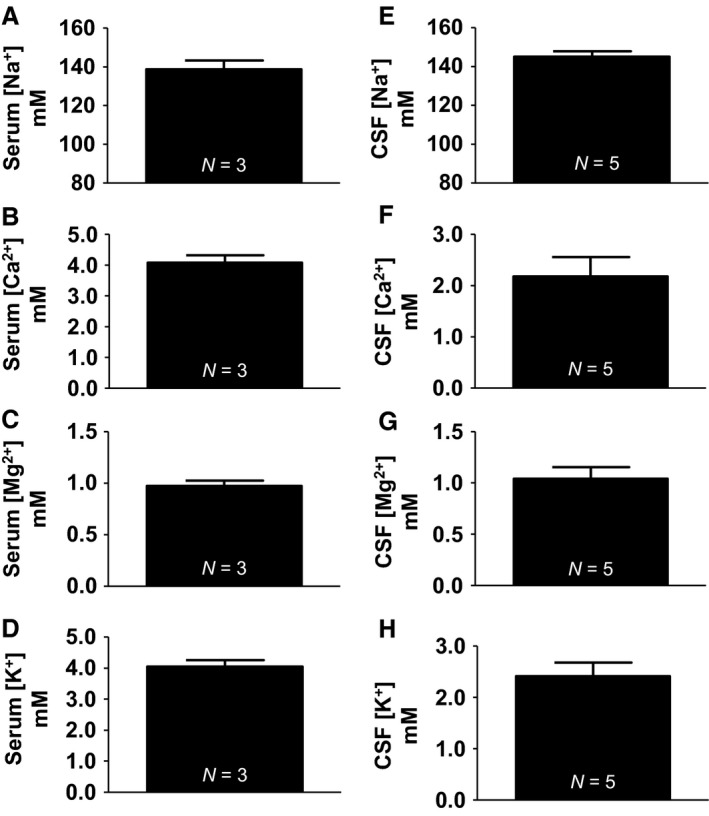
Cation concentrations from serum and CSF. (A) Serum sodium (138.8 ± 4.56 mmol/L). (B) Serum potassium (4.05 ± 0.21 mmol/L). (C) Serum calcium (4.07 ± 0.26 mmol/L). (D) Serum magnesium (0.98 ± 0.05 mmol/L). (E) CSF sodium (145.1 ± 2.81 mmol/L). (F) CSF potassium (2.41 ± 0.26 mmol/L). (G) CSF calcium (2.18 ± 0.38 mmol/L). (H) CSF magnesium (1.04 ± 0.11 mmol/L).

Using ion chromatography, we analyzed liquid samples; serum and CSF obtained from Sprague‐Dawley rats for the concentration of cations (Na^+^, K^+^, Ca^2+^, and Mg^2+^). Serum concentration of cations (Na^+^, K^+^, Ca^2+^, and Mg^2+^) were in mmol/L: 138.8 ± 4.56, 4.05 ± 0.21, 4.07 ± 0.26, 0.98 ± 0.05 (Fig. 4). CSF concentration of cations (Na^+^, K^+^, Ca^2+^, and Mg^2+^) were in mmol/L: 145.1 ± 2.81, 2.41 ± 0.26, 2.18 ± 0.38, 1.04 ± 0.11 (Fig. 4). Serum and CSF electrolyte ranges obtained from IC are also reported (Table 1).

**Table 1:** Ranges of electrolytes in serum and cerebrospinal fluid (CSF)

Ion source	Na^+^	K^+^	Ca^2+^	Mg^2+^	Cl^−^	^−^OAc
NS (*N* = 6)[mmol/L]	139.6–155.2	1.86–3.29	1.4–3.3	0.84–1.47	113.9–126.9	0.07–0.33
NS Range (*N* = 6) [mmol/L]	131.2–147.0	3.73–4.44	3.57–4.45	0.91–1.08	92.6–104.2	0.12–0.37

**Figure 5:**
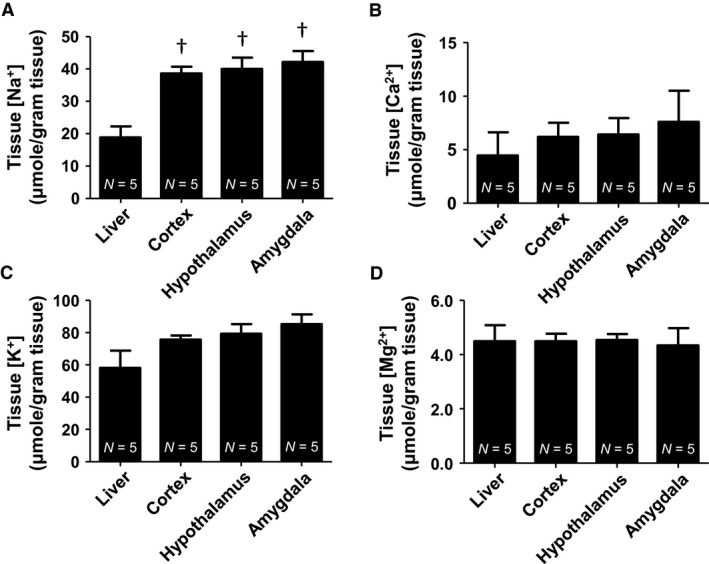
Cation concentrations from tissue samples. (A) Tissue Na^+^ (*μ*mol/gram tissue): Liver (18.86 ± 3.42), cortex (38.58 ± 2.09), hypothalamus (39.98 ± 3.54), and amygdala (42.16 ± 3.37). (B) Tissue K^+^ (*μ*mol/gram tissue): Liver (58.14 ± 10.66), cortex (75.62 ± 2.56), hypothalamus (79.22 ± 5.97), and amygdala (85.21 ± 6.08). (C) Tissue Ca^2+^ (*μ*mol/gram tissue): Liver (4.45 ± 2.16), cortex (6.20 ± 1.32), hypothalamus (6.41 ± 1.55), and amygdala (7.59 ± 2.90). (D) Tissue Mg^2+^ (*μ*mol/gram tissue): Liver (4.49 ± 0.59), cortex (4.49 ± 0.28), hypothalamus (4.53 ± 0.22), and amygdala (4.34 ± 0.63). (†vs. control one‐way ANOVA.

Next we prepared, extracted, and analyzed tissue samples from the liver, cortex, hypothalamus, and amygdala for cation concentrations. Tissue Na^+^ (*μ*mol/gram tissue): Liver (18.86 ± 3.42), cortex (38.58 ± 2.09), hypothalamus (39.98 ± 3.54), and amygdala (42.16 ± 3.37). Tissue samples from the cortex, hypothalamus, and amygdala were significantly (*P* < 0.05) higher in sodium concentration compared to the peripheral liver tissue (Fig. 5A). Tissue K^+^ (*μ*mol/gram tissue): Liver (58.14 ± 10.66), cortex (75.62 ± 2.56), hypothalamus (79.22 ± 5.97), and amygdala (85.21 ± 6.08). There was no statistical difference in any tissue regions measured for potassium concentrations (Fig. 5C). Tissue Ca^2+^ (*μ*mol/gram tissue): Liver (4.45 ± 2.16), cortex (6.20 ± 1.32), hypothalamus (6.41 ± 1.55), and amygdala (7.59 ± 2.90). There was no statistical difference in any tissue region measured for calcium concentration (Fig. 5B). Tissue Mg^2+^ (*μ*mol/gram tissue): Liver (4.49 ± 0.59), cortex (4.49 ± 0.28), hypothalamus (4.53 ± 0.22), and amygdala (4.34 ± 0.63). There was no statistical difference in any tissue region measured for magnesium concentration (Fig. 5D). Tissue electrolyte ranges obtained from IC are also reported (Table 2).

**Table 2:** Ranges of electrolytes in tissue

Source Ion	Liver [*μ*mol/gram tissue]	Cortex [*μ*mol/gram tissue]	Hypothalamus [*μ*mol/gram tissue]	Amygdala [*μ*mol/gram tissue]
Na^+^	9.92–28.74	32.03–44.61	34.16–52.55	33.63–55.24
K^+^	26.6–84.70	68.4–83.2	66.6–97.5	73.7–108.2
Ca^2+^	0.19–12.18	2.97–10.43	3.30–11.42	2.13–18.87
Mg^2+^	2.57–5.76	3.78–5.38	3.97–35.92	2.14–5.85
Cl^−^	11.52–27.76	23.48–27.93	25.55–35.92	25.63–33.41
^−^OAc	0.43–1.46	1.98–4.70	2.38–5.77	1.94–5.65

### Chloride and acetate standard curve and representative IC chromatogram

**Figure 6:**
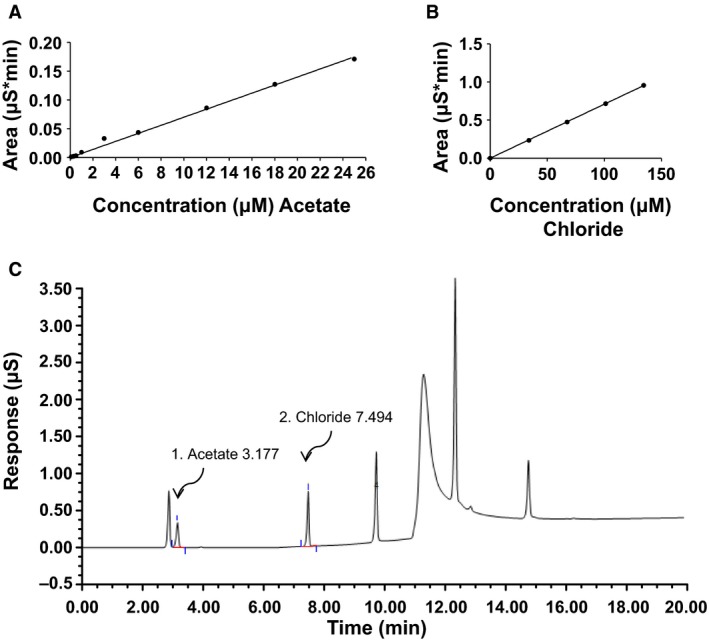
Anion standard curves and representative chromatogram. (A) Acetate standard curve. (B) Chloride standard curve. (C) Representative anion standard chromatogram from IC. Anion peaks were well resolved. A linear regression line was fitted to each standard curve and unknown anion concentrations determined from the slope of the line.

Standard curves for both chloride (Fig. 6B) and acetate (Fig. 6A) were constructed and a linear regression line fit for each anion. Figure 6C is a representative IC chromatogram for chloride and acetate. Peaks were well resolved and elution and detection of acetate and chloride occurred at (min): 3.177 and 7.494, respectively (Fig. 6C). Figure 8B is a representative IC chromatogram from a cortex tissue sample of a Sprague‐Dawley rat. Acetate and chloride elution and detection occurred at (min): 3.161 and 7.491, respectively (Fig. 8B).

### Chloride and acetate concentrations in liquid and tissue samples

**Figure 7:**
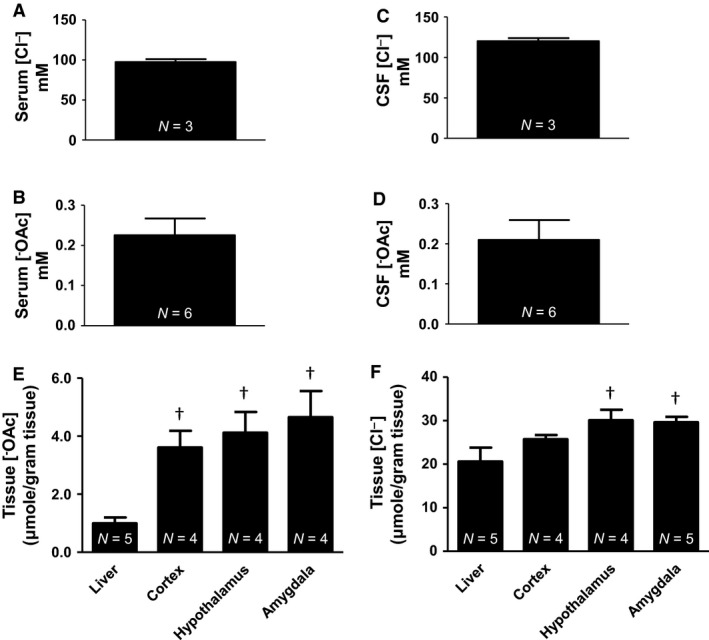
Summary data for anions. (A) Serum Cl^−^ (97.7 ± 3.42 mmol/L). (B) Serum ^−^ OAc (0.23 ± 0.04 mmol/L). (C) CSF Cl^−^ (120.2 ± 2.81 mmol/L). (D) CSF ^−^ OAc (0.21 ± 0.05 mmol/L). (E) Tissue ^−^ OAc (*μ*mol/gram tissue): Liver (1.00 ± 0.19), cortex (3.61 ± 0.58), hypothalamus (4.12 ± 0.72), and amygdala (4.65 ± 0.90). (F) Tissue Cl^−^ (*μ*mol/gram tissue): Liver (20.57 ± 3.20), cortex (25.72 ± 0.95), hypothalamus (30.09 ± 2.40), and amygdala (29.61 ± 1.25). (†vs. liver one‐way ANOVA).

Using ion chromatography we analyzed liquid samples, serum and CSF for chloride and acetate concentrations. Serum contained chloride and acetate (mmol/L): 97.7 ± 3.42 and 0.23 ± 0.04, respectively (Fig. 7A and B). CSF contained chloride and acetate (mmol/L): 120.2 ± 3.75, 0.21 ± 0.05, respectively (Fig. 7C and D).

Similarly to the cation measurements in tissue, we analyzed the concentration of chloride and acetate in tissue samples of the liver, cortex, hypothalamus, and amygdala. Tissue Cl^−^ (*μ*mol/gram tissue): Liver (20.57 ± 3.20), cortex (25.72 ± 0.95), hypothalamus (30.09 ± 2.40), and amygdala (29.61 ± 1.25). Tissue chloride concentration was significantly (*P* < 0.05) higher in the hypothalamus and amygdala compared to the peripheral liver tissue (Fig. 7F). Tissue ^−^OAc (*μ*mol/gram tissue): Liver (1.00 ± 0.19), cortex (3.61 ± 0.58), hypothalamus (4.12 ± 0.72), and amygdala (4.65 ± 0.90). Tissue acetate concentrations were significantly (*P* < 0.05) higher in the cortex, hypothalamus, and amygdala compared to the peripheral liver tissue (Fig. 7E).

### Method validation with ion selective electrode

**Figure 8:**
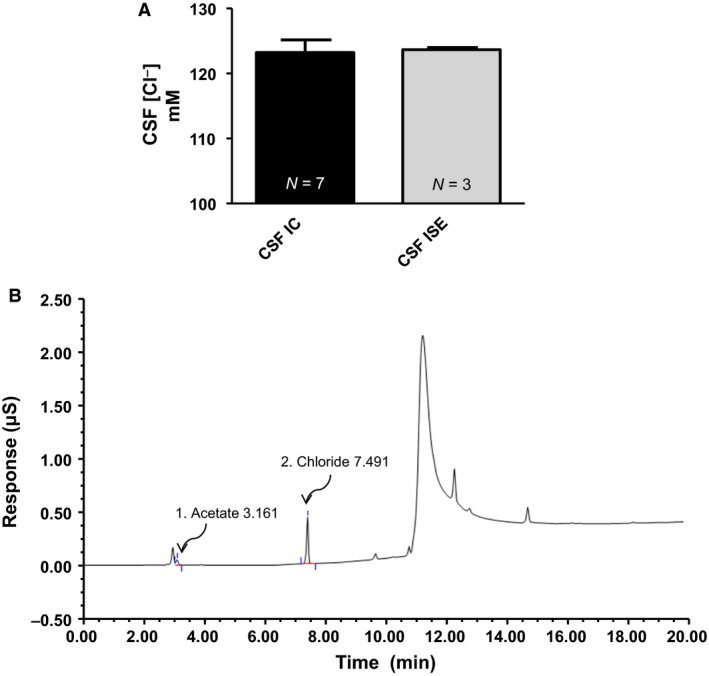
Method validation between IC and ISE. (A) Summary data for CSF chloride concentrations measured with IC (123.2 ± 1.94 mmol/L) and ISE (123.7 ± 0.33 mmol/L). There was no statistical difference between CSF chloride concentrations from IC vs. ISE. (B) Representative anion chromatogram from IC for a tissue sample from the cortex of a Sprague‐Dawley rat.

To validate the use of IC for the measurement of cations and anions, including acetate from various physiological samples, we compared CSF chloride concentrations from IC with a known medical diagnostic machine which used a chloride selective electrode (ISE). CSF chloride concentrations measured using IC had a mean value of 123.2 ± 1.94 mmol/L (*n* = 7) and had no statistical difference between ISE 123.7 ± 0.33 (*n* = 3) (Fig. 8A).

### Cation and anion concentrations in urine from normal salt\fed rats

To verify the application of our method in urine, we analyzed the urine of NS fed rats. Normal salt (NS) rats had urine concentrations of Na^+^, K^+^, Ca^2+^, Mg^2+^, Cl^−^, and ^–^OAc (mmol/L): 98.8 ± 7.7, 184.8 ± 20.2, 0.87 ± 0.08, 1.88 ± 0.3, 103.3 ± 7.9, and 6.1 ± 1.3 (Table 3).

**Table 3:** Urine electrolyte concentrations from NS rats

Ion source	Na^+^	K^+^	Ca^2+^	Mg^2+^	Cl^−^	^−^OAc
NS (*N* = 6)[mmol/L]	98.8 ± 7.7	184.8 ± 20.2	0.87 ± 0.08	1.88 ± 0.3	103.3 ± 7.9	6.1 ± 1.3
NS Range (*N* = 6) [mmol/L]	67.9–117.6	131.3–243.5	0.7–1.24	1.02–2.90	77.5–124.5	3.6–10.5

NS, normal salt (0.4%) NaCl.

## Discussion

We first analyzed cations from our liquid samples; serum and CSF and compared those to the values reported previously in literature. For cations, Na^+^ and K^+^ are the most commonly measured in both serum and CSF. Older publications report Na^+^ and K^+^ concentrations in serum of 148.4 ± 0.8 mmol/L and 3.16 ± 0.04 mmol/L, respectively (Reed et al. 1967), 134.2 mmol/L and 4.3 mmol/L (Hald 1947), both analyzed via flame photometry. Newer analytical methods have since emerged; Gomes and colleagues exploring high‐salt diet and the development of hypertension on weaning rats utilized flame photometry to measure Na^+^ and K^+^ concentrations from plasma and CSF. Plasma Na^+^ reported was around 143 mmol/L, plasma K^+^ around 4.7 mmol/L, CSF Na^+^: 147.8 ± 4.8 mmol/L and CSF K^+^: 2.85 ± 0.14 mmol/L (Gomes et al. 2017). Stocker and colleagues exploring hypernatremia utilized a hand held commercially available device (I‐STAT) to measure CSF Na^+^, with baseline sodium at 155.3 ± 0.3 mmol/L (Stocker et al. 2015). Consistent with these previous publications, our method replicates values reported in the literature. Our serum Na^+^ and K^+^ were: 138.8 ± 4.56 mmol/L and 4.05 ± 0.21 mmol/L, respectively. Likewise, CSF concentrations of Na^+^ and K^+^ were: 145.1 ± 2.81 mmol/L and 2.41 ± 0.26 mmol/L, respectively.

Tissue concentrations of Na^+^ and K^+^ are reported far less than liquid samples, probably owning to the relative difficulty in current analysis techniques. Christensen and colleagues utilized 23Na and MRI to quantify brain tissue concentrations relative to an older technique using radiolabeled, 22Na (Christensen et al. 1996). Reported brain tissue concentrations for 23Na and 22Na were 45 ± 4 mmol/L and 49 ± 6 mmol/L (Christensen et al. 1996). Yushmanov's group also reported values of Na^+^ in brain, 45.9 ± 0.9 mEq/kg tissue, and 27.4 mEq/kg tissue in the liver (Yushmanov et al. 2007). Our reported tissue Na^+^ (*μ*mol/gram tissue): Liver (18.86 ± 3.42), cortex (38.58 ± 2.09), hypothalamus (39.98 ± 3.54), and amygdala (42.16 ± 3.37) closely compares to these previously reported values. For brain tissue K^+^, Yushmanov and colleagues reported values for brain and liver tissue of 70 ± 1 mEq/kg tissue and 43 mEq/kg tissue, ~70 and 43 *μ*mol/gram tissue (Yushmanov et al. 2007). Our reported brain and liver tissue K^+^ concentrations from our extraction and IC method were: (*μ*mol/gram tissue): Liver (58.14 ± 10.66), cortex (75.62 ± 2.56), hypothalamus (79.22 ± 5.97), and amygdala (85.21 ± 6.08), which are in agreement with these values.

Ca^2+^ and Mg^2+^are much less reported cations in serum and CSF. Plasma and CSF Ca^2+^ reported by Jones and Keep were 2.5 mmol/L and 1.4 mmol/L, respectively (Jones and Keep 1988). Watchon reported serum Ca^2+^ and Mg^2+^ of 11.92 mg/100 mL and 4.43 mg/100 mL, equivalent to 2.97 mmol/L and 1.90 mmol/L (Watchorn 1933). Their range of Ca^2+^ concentrations was 9.61–14.04 mg/100 mL, ~2.41–3.52 mmol/L. Furthermore, a more recent publication from Dodge and colleagues found mice had a mean serum Ca^2+^ concentrations of 4.72 mmol/L (Dodge et al. 2013). Dodge's group reports the use of two instruments, VetACE clinical chemistry system (Alpha Wassermann) for calcium and phosphate and Beckman CX5 clinical chemistry analyzer (Beckman Coulter) which uses indirect ISE for other electrolytes. Our values obtained from IC are also consistent with the Ca^2+^ and Mg^2+^ reported in serum and CSF: 4.07 ± 0.26 mmol/L and 0.98 ± 0.05 mmol/L, respectively for serum and 2.18 ± 0.38 mmol/L and 1.04 ± 0.11 mmol/L, respectively for CSF. Much of the Mg^2+^ in the brain and liver is primarily bound with ATP as reported by Veloso and colleagues. Reported values of insoluble, soluble and ATP bound Mg^2+^ from the brain in (*μ*mol/gram tissue) were: 2.2, 1.2, and 1.6, respectively (Veloso et al. 1973). The summation of tissue bound Mg^2+^ in the brain is around 5 *μ*mol/gram tissue. Liver values in (*μ*mol/gram tissue) were: 2.2, 3.5, and 2.5, respectively, with a total of around 8.2 *μ*mol/gram tissue (Veloso et al. 1973). Our values of tissue bound Mg^2+^ closely agrees with these values. Tissue Mg^2+^ (*μ*mol/gram tissue): Liver (4.49 ± 0.59), cortex (4.49 ± 0.28), hypothalamus (4.53 ± 0.22), and amygdala (4.34 ± 0.63), although liver is slightly lower than those reported by Veloso and colleagues. Our reported tissue Ca^2+^ (*μ*mol/gram tissue): Liver (4.45 ± 2.16), cortex (6.20 ± 1.32), hypothalamus (6.41 ± 1.55), and amygdala (7.59 ± 2.90). These are similar, albeit slightly higher than those reported by Rappaport in an ischemia investigation. Brain tissue Ca^2+^ in unoperated control groups was 2.41 ± 0.16 *μ*mol/gram tissue as analyzed by atomic absorption (Rappaport et al. 1987).

**Figure 9:**
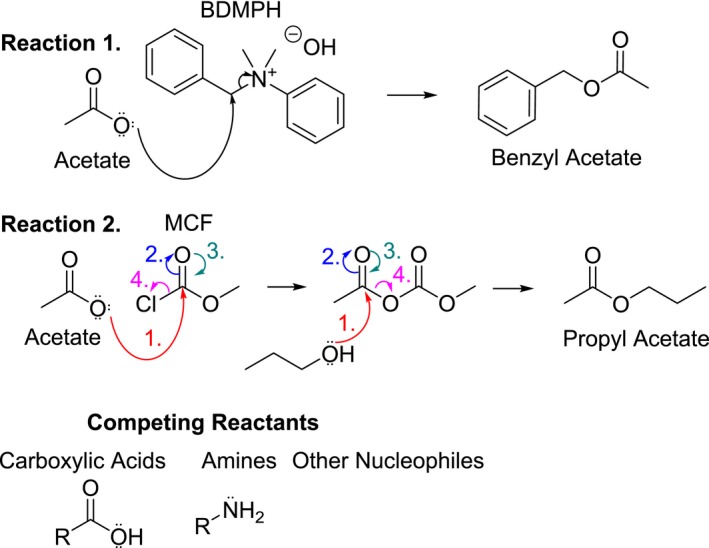
Known ester derivatization reactions with acetate. Reaction 1 shows acetate derivatization to benzyl acetate using benzyldimethylammonium hydroxide as an ester derivatizing agent.(Richards et al. 1975) Reaction 2 shows acetate derivatization to propyl acetate using methylchloroformate as a derivatizing agent, followed by a subsequent reaction with propyl alcohol.(Tumanov et al. 2016) Any amino acids, carboxylic acids or nucleophiles remaining in samples are capable of reacting with derivatizing reagents and forming side products, drastically reducing the apparent concentration of SCFA and or leading to wide ranges for reported values. BDMPH, (benzyldimethylphenylammonium hydroxide; MCF, methylchloroformate.

Our group previously published ethanol and acetate microinjection data for ethanol induced hypertension (Chapp et al. 2014) and quickly realized the reliable measurement of acetate concentrations was going to be inherently important for our continued research. Traditional derivatization techniques appeared to yield variability, at least partially due to the method employed and possible side reactions (Fig. 9). To combat this, we implemented a complete aqueous extraction, absent of any strong acids/bases, or organic solvent extractions. For the analysis of ^−^OAc and Cl^−^, we found that ^−^OAc concentration in serum and CSF were: 0.23 ± 0.04 mmol/L and 0.21 ± 0.05 mmol/L, respectively and chloride concentration in serum and CSF were: 97.7 ± 3.42 mmol/L and 120.2 ± 3.75 mmol/L, respectively (Fig. 7A and B). Tissue Cl^−^ (*μ*mol/gram tissue): Liver (20.57 ± 3.20), cortex (25.72 ± 0.95), hypothalamus (30.09 ± 2.40), and amygdala (29.61 ± 1.25). Tissue chloride concentration was significantly (*P* < 0.05) higher in the hypothalamus and amygdala compared to the peripheral liver tissue (Fig. 7F). Tissue ^−^OAc (*μ*mol/gram tissue): Liver (1.00 ± 0.19), cortex (3.61 ± 0.58), hypothalamus (4.12 ± 0.72), and amygdala (4.65 ± 0.90). Tissue acetate concentrations were significantly (*P* < 0.05) higher in the cortex, hypothalamus and amygdala compared to the peripheral liver tissue (Fig. 7E). To provide proof of concept, we compared CSF chloride concentrations from IC with an ion selective electrode method used for medical diagnostics. There was no significant differences between CSF IC versus CSF ISE; 123.20 ± 1.94 and 123.70 ± 0.33 mmol/L, respectively (Fig. 8A).

Literature values reported for Cl^−^ concentrations in rat CSF vary, with previous concentrations measured at 117.3 mmol/L (Reed et al. 1967) 116.4 ± 0.5 mmol/L (Stocker et al. 2015) and another reporting a range of 110–125 mmol/L (Amtorp and Sorensen 1974). Similarly, Cl^−^ concentration from CSF in humans was reported at 123.2 ± 2.62 mmol/L (Pye and Aber 1982). Serum concentrations reported from rats had an average of 99 mmol/L,(Ralli et al. 1941) and SD rats from Charles River reported an average range of 100–105 mmol/L. Human serum Cl^−^ concentrations range from 96 to 106 mmol/L. Thus, our Cl^−^ concentrations measured by IC align with those reported for both rats and humans. Furthermore, the values obtained in CSF from IC were nearly identical with those obtained through an ion selective electrode used for medical diagnostics (Fig. 8A), verifying the validity of the method.

Acetate concentrations are far less common in the literature, with older publications reporting values (mean ± SD) of 1.117 ± 0.079 *μ*mol/gram liver tissue and 10.8 ± 0.89 *μ*mol/100 mL serum, ~0.11 mmol/L (Richards et al. 1975). An NMR spectroscopy study reported baseline plasma ^−^OAc concentration of 0.19 ± 0.05 mmol/L (Deelchand et al. 2009) similar to our reported serum value of 0.23 ± 0.04 mmol/L (Fig. 7B). Furthermore, a recent publication following a traditional derivatization method for acetate (Turnbaugh et al. 2006) reported liver and hypothalamus concentrations of 0.9 ± 0.2 and 1.9 ± 0.1 *μ*mol/gram tissue, respectively (Perry et al. 2016). Our reported acetate tissue values are also consistent with these findings, albeit slightly higher in brain tissue. Tissue ^−^OAc (*μ*mol/gram tissue): Liver (1.00 ± 0.19), cortex (3.61 ± 0.58), hypothalamus (4.12 ± 0.72), and amygdala (4.65 ± 0.90).

To include one more physiological liquid sample, we also analyzed urine electrolytes from NS fed rats. Urine electrolyte concentrations typically have a large reported range which is dependent on several factors, but the most prominent is water/food and salt intake (Foss et al. 2013; Gomes et al. 2017; Osborn et al. 2014; Wang et al. 2016; Yu et al. 2016). As such, most research groups measure 24 h sodium and water balance to help determine water and sodium intake, urine volume and urine sodium excretion (Foss et al. 2013; Gomes et al. 2017; Osborn et al. 2014; Wang et al. 2016; Yu et al. 2016). Non‐24 h urine analysis though can provide insight into progression of disease, including electrolyte imbalance and acid/base disorders (Gray et al. 2013; Hamm et al. 2015; Waldrop 2008).

Reported ranges and concentrations of urine electrolytes (Na^+^, K^+^, Ca^2+^, Mg^2+^, and Cl^−^) from SD rats vary and have been reported from other groups. Shevok and colleagues reported numerous electrolytes, range and concentration (mean ± SE), respectively (mmol/L); Na^+^: 45–250, 149.0 ± 60, K^+^: 120–475, 267 ± 99, Ca^2+^: 0.42–10.02, 3.39 ± 2.14 and Mg^2+^: 7.2–37.6, 21.4 ± 5.7 (Shevock et al. 1993). Our values (Table 3.) fall within the ranges reported, other than Ca^2+^ and Mg^2+^ being lower than reported by Shevok's group (Shevock et al. 1993). There was no significant difference in acetate excretion between groups, and to the best of our knowledge, we are the first to report on urine acetate concentrations in SD rats.

### Advantages

IC offers a series of advantages over more traditional methods for cation, anion, and small organic ion quantification. First, for small organic ions such as acetate, the common method utilizes derivatizations to esters that can have large variability due to possibility of side reactions and reduced yield from organic extractions (Fig. 9). Our method eliminates all of these issues because of our complete aqueous workup and extraction. Furthermore, with regard to acetate contamination, sonication of centrifuge tubes, fresh water and repeated wash steps on syringes and filters reduced acetate to undetectable levels which drastically reduces the variability and background contamination reported previously by other groups. Another advantage is with dual detection systems, most cations and anions are capable of being measured at once per sample. This substantially increases the amount of available data a research group has to work with, when looking at electrolyte imbalances in pathophysiological states and reduces the volume of sample required for the ion concentrations. Another benefit is the detection range. We attempted to keep our diluted samples in a range between 0.5 and 400 *μ*mol/L, thus there is flexibility for the researcher in dilution factors depending on the ion of interest.

### Disadvantages

The IC system is an expensive setup, and requires some training to be able to execute methods for given cation and anion quantification. That being said, most major academic research universities should have an IC system on campus with at least one well‐trained individual capable of assisting. The IC method also takes time for processing samples, as great accuracy is required in dilutions, often requiring attention to detail and good analytical techniques. Furthermore, additional time is required to clean tubes, syringes, and filters. Run times per sample are also significantly longer compared to hand held and clinical/commercially available devices. Typical sample runtimes are ~20 min per injection, usually at least two injections per sample and a 20 min water clear at the end between each sample. While this initially appears long, the amount of information gathered if using dual ion detection far outweighs the longer runtimes in our opinion.

## Perspective

In summary, we conclude that measurement of cation and anion concentrations, including acetate from physiological samples using IC is an accurate, reliable method with advantages over traditional methodologies. Depending on the researchers need, this method may offer more comprehensive information compared to commonly used methods for ion concentration measurements. Furthermore, IC units equipped with a splitter for dual ion detection (cations/anions including, small SCFA) provides researchers a means to quantify common ions and difficult SCFA which are altered in pathophysiological states (Christensen et al. 1996; Comerford Sarah et al. 2014; Drapeau and Nachshen 1988; Jiang et al. 2013; Mashimo et al. 2014; Perry et al. 2016; Rappaport et al. 1987; Singh et al. 2016; Stocker et al. 2015; Watchorn 1933; Yang et al. 2006) with minimal sample volume. We anticipate that this new method will be useful to many research groups investigating SCFA and electrolyte concentrations and those effects on: water and electrolyte homeostasis, stroke, cardiovascular disease, hypertension, metabolic processes, cancer, and ethanol metabolism (Chapp et al. 2014), to name a few. Samples included for analysis can vary, but are not anticipated to be limited to: CSF (Gomes et al. 2017), blood (Gomes et al. 2017; Stocker et al. 2015), tissue, cell culture, urine (Foss et al. 2013; Lohmeier et al. 2005; Osborn et al. 2012; Schreihofer et al. 1999) and feces. Additionally, complete aqueous extraction removes the need for organic solvents (Richards et al. 1975), strong acids/bases (Kamphorst et al. 2014; Richards et al. 1975; Turnbaugh et al. 2006) and ester derivatizing agents (Kamphorst et al. 2014; Richards et al. 1975; Turnbaugh et al. 2006) in SCFA analysis which we envision will drastically increase the reproducibility, and accuracy while reducing variability.

## Conflict of Interest

There are no conflicts of interests.
